# Analysis of differential effects of host plants on the gut microbes of *Rhoptroceros cyatheae*

**DOI:** 10.3389/fmicb.2024.1392586

**Published:** 2024-06-19

**Authors:** Bingchen Zhang, Weicheng Yang, Qinqin He, Hangdan Chen, Bingjie Che, Xiaojie Bai

**Affiliations:** ^1^School of Life Sciences, Guizhou Normal University, Guiyang, Guizhou, China; ^2^Guizhou Chishui Alsophila National Nature Reserve Administration Bureau, Chishui, Guizhou, China

**Keywords:** Hymenoptera, intestinal microbes, relict plant, hosts diets, adaptability, high-throughput sequencing

## Abstract

As an indispensable part of insects, intestinal symbiotic bacteria play a vital role in the growth and development of insects and their adaptability. *Rhoptroceros cyatheae*, the main pest of the relict plant *Alsophila spinulosa*, poses a serious threat to the development of the *A. spinulosa* population. In the present study, 16S rDNA and internal transcribed spacer high-throughput sequencing techniques were used to analyze the structure of intestinal microbes and the diversity of the insect feeding on two different plants, as well as the similarities between the intestinal microorganisms of *R. cyatheae*. The dominant bacteria of leaf endophytes were also compared based on the sequencing data. The results showed that Proteobacteria, Firmicutes, and Actinobacteria were the dominant phyla of intestinal bacteria, and Ascomycota was the dominant phylum of intestinal fungi. *Allorhizobium-Neorhizobium-Pararhizobium-Rhizobium*, *Methylobacterium-Methylorubrum*, and *Enterococcus* were the dominant genera in the intestine of *R*. *cyatheae* feeding on two plants, and the relative abundance was significantly different between the two groups. *Candida* was the common dominant genus of intestinal fungi in the two groups, and no significant difference was observed in its abundance between the two groups. This showed that compared with the intestinal fungi of *R*. *cyatheae*, the abundance of the intestinal bacteria was greatly affected by food. The common core microbiota between the microorganisms in *A. spinulosa* leaves and the insect gut indicated the presence of a microbial exchange between the two. The network correlation diagram showed that the gut microbes of *R*. *cyatheae* feeding on *Gymnosphaera metteniana* were more closely related to each other, which could help the host to better cope with the adverse external environment. This study provides a theoretical basis for the adaptation mechanism of *R*. *cyatheae* and a new direction for the effective prevention and control of *R*. *cyatheae*.

## 1 Introduction

The tree fern *Alsophila spinulosa* is a world-famous relict plant and is currently on the red list of threatened species by the International Union for Conservation of Nature (Ma et al., 2020). *Rhoptroceros cyatheae* (Hymenoptera: Selandriidae) mainly harms *A. spinulosa* and *Gymnosphaera metteniana*. Adult females of *R. cyatheae* lay eggs on the leaves of *A. spinulosa* saplings, and their larvae primarily feed on the mesophyll tissue. In severe cases, these larvae can consume the leaves of the entire plant, thus markedly affecting the photosynthesis and spore reproduction of *A. spinulosa* (Xu et al., 2021).

Plant tissues often produce many indigestible and toxic substances during growth and development; consequently, herbivorous insects have evolved a series of strategies to adapt to different plants, including working with their symbionts to absorb nutrients (Salem et al., 2014; Shikano, 2017; Mason et al., 2019a; McMillan, 2023). With the development of high-throughput technology, the contribution of intestinal symbiotic bacteria to host digestion and absorption (Engel and Moran, 2013; Jing et al., 2020), detoxification (Blanton and Peterson, 2020; Siddiqui et al., 2022), growth and development (Yang et al., 2022), and oviposition induction (Qiao et al., 2019) has been supported by extensive data. The gut microbiome of herbivorous insects is an important part of the insect–plant interaction, which is mainly manifested in two major functions of the gut microbiome as follows: supplementing nutrients and degrading toxic secondary metabolites (Hammer and Bowers, 2015; Pérez-Cobas et al., 2015). For example, the gut microbiome provides essential amino acids, vitamins, carbon, and nitrogen to the host, which ensures the normal growth and development of the insects feeding on unsuitable plants (Scully et al., 2013; Ayayee et al., 2014; Salem et al., 2014). The intestinal microbes of these insects can reduce the damage caused by the secondary metabolites of plants, such as tannins, caffeine, and nicotine, so that the insect can better digest the feeding plants (Berasategui et al., 2017; Zhang et al., 2020). In addition, gut microbes affect the growth and development, defense of natural enemies, mating, reproduction, and other aspects of these insects (Leftwich et al., 2018; Duplais et al., 2021).

The composition of gut microbial communities can be driven by various factors, including intestinal structure (Chen et al., 2016), geography and climate change (Luo et al., 2020), diet, and other factors (Yun et al., 2014). The specificity of the insects and its developmental stages are important factors affecting the intestinal communities. For example, the gut microbes of cockroaches and termites feeding on wood were different (Liu et al., 2020). Gao et al. (2019) found that Firmicutes were dominant in the gut of the third and fifth instar larvae of *Spodoptera exigua*, whereas Proteobacteria were dominant in other stages. Studies on *Apis mellifera* and *Plutella xylostella* have found that geographical locations and climatic changes are the factors affecting intestinal microbial composition (Ludvigsen et al., 2015; Kaur et al., 2022). Diet is the dominant factor associated with the changes in intestinal microorganisms, and food types can rapidly and greatly alter the intestinal microbial communities of insects (Ana et al., 2015). This dynamic change in intestinal microorganisms is one of the reasons why insects can successfully feed on different host plants (Strano et al., 2018; Zhang et al., 2022). Food can directly interact with the intestine after the insect has fed on the plant. Moreover, plant materials and endogenous microorganisms are the main force in shaping the intestinal microorganisms of insects (Hardoim et al., 2015; Bozorov et al., 2019; Chen et al., 2020; Xiong, 2022; Pirttilä et al., 2023). Therefore, investigating the structure of intestinal microbial communities in insects and the role of food in shaping this intestinal microbiome is crucial. At present, research on the effect of food on the intestinal symbiotic bacteria of *R*. *cyatheae* is lacking. Thus, in the present study, we compared the effects of food on the gut microbiota of *R*. *cyatheae* by analyzing the structure of endophytic bacteria in the leaves of two host plants. We also analyzed the diversity of gut microbiota of *R*. *cyatheae* fed with two different host plants during its peak occurrence period (namely May–June) in a year. This study aimed to assess the effects of host plants on *R*. *cyatheae* and to provide a basis for the development of efficient and green control measures against the insect.

## 2 Materials and methods

### 2.1 Sample collection

*Rhoptroceros cyatheae* was obtained from the Guizhou Chishui *Alsophila* National Nature Reserve (28°25′12″N, 106°01′03″E) in May 2023. All larvae collected from the forest were fed in an artificial climate incubator (SPX-280, Ningbo Jiangnan Instrument Factory, China; 26 ± 1°C, 75% ± 10% relative humidity, and 16L:8D photoperiod). Newly hatched larvae were reared with *A. spinulosa* and *G. metteniana* until the fifth instar. These two plants were obtained from the Chishui *Alsophila* National Nature Reserve. After 24 h of starvation, the larvae were sterilized with 75% ethanol for 1 min and then washed thrice with sterile water. The complete intestinal tract was dissected in sterile phosphate-buffered saline under a stereoscope, and 15–20 intestinal tracts were mixed as a biological replication, with three replicates per treatment (As: feeding on *A. spinulosa*; Gm: feeding on *G. metteniana*). The larvae were identified as the fifth instar according to their molting times (Xu et al., 2021). A sterile 15-mm sample puncher was used to collect samples from fresh and intact *A. spinulosa* leaves (AsL) and *G. metteniana* leaves (GmL) into a sterile culture plate, and 75% ethanol was used to clean the surface. The samples were mixed and ground under liquid nitrogen for subsequent DNA extraction.

### 2.2 DNA extraction and PCR amplification

The total DNA of the samples was extracted as for the E.Z.N.A^®^ soil DNA Kit (Omega Bio-tek, Norcross, GA, USA) instructions. The primers 799F (5′-AACMGGATTAGATACCCKG-3′)/1193R (5′ACGTCATCCCCACCTTCC-3′) and ITS1F (5′-CTTGGTCATTTAGAGGAAGTAA-3′)/ITS2R (5′-GCTGCGTTCTTCATCGATGC-3′) were used for PCR. PCR cycling conditions were as follows: 5 min at 94°C for initialization, 30 cycles of denaturation for 30 s at 95°C, 30 s of annealing at 55°C, and 30 s of extension at 72°C, followed by a 10-min final elongation at 72°C. The PCR product was extracted by performing 2% agarose gel electrophoresis and purification with PCR Clean-Up Kit (YuHua, Shanghai, China) according to the manufacturer’s instructions and quantified using Qubit 4.0 (Thermo Fisher Scientific, USA). NEXTFLEX^®^ Rapid DNA-Seq Kit (YuHua, Shanghai, China) was used to construct a library of purified PCR products.

### 2.3 Quality control and operational taxonomic unit (OTU) identification

Fastp^1^ software was used to perform quality control on the double-ended original sequence, and FLASH^2^ software was used for splicing: (i) The reads were truncated at any site receiving an average quality score of <20 over a 50 bp sliding window, the truncated reads shorter than 50 bp were discarded, and the reads containing ambiguous characters were also discarded; (ii) Only overlapping sequences longer than 10 bp were assembled according to their overlapped regions. The maximum mismatch ratio of the overlap region was 0.2. Reads that could not be assembled were discarded; (iii) The samples were distinguished according to their barcodes and primers, and the sequence direction was adjusted, the exact barcode was matched; two nucleotides were mismatched in primer matching. Then, the optimized sequences were clustered into operational taxonomic units (OTUs) using UPARSE (version 7.1) with 97% sequence similarity. The OTU table was manually filtered, and chimeric, chloroplast, and mitochondrion sequences were removed. RDP Classifier (version 2.11) was used to compare each OTU representative sequence with those in the database Silva and Unite, and the confidence threshold was 70%.

### 2.4 Diversity analysis

Alpha and beta diversity indices were estimated at the OTU level using MOTHUR and UniFrac, respectively. The Kruskal–Wallis test was used to analyze the α-diversity index of bacteria and fungi in the samples. The Wilcoxon signed-rank test and T-test (false discovery rate correction) were used for pairwise comparison. The principal coordinate analysis was performed based on the Bray–Curtis distance, and the Adonis test was used to analyze the difference between the groups, with *p* < 0.05 considered statistically significant. The linear discriminant analysis (LDA) effect size^3^ was calculated to identify the significantly abundant taxa (phylum to genera) of bacteria among the different groups (LDA > 4, *p* < 0.05). To understand the relationships among the genera, Spearman’s correlation coefficients were used for network analyses (Networkx: version 1.11). Network topological properties were calculated using Gephi. PICRUSt2^4^ and FAPROTAX (1.2.1) were used to predict the function of the microbial community in different samples.

## 3 Results

### 3.1 Annotation and evaluation of sequences

Illumina Miseq sequencing was performed to characterize the 16S rDNA and ITS2 regions of the leaves and *R*. *cyatheae* gut. A total of 831,798 bacterial reads (Supplementary Table S1) with an average length of 376 bp were obtained. Additionally, 1,074,630 fungal reads with an average length of 251 bp (Supplementary Table S2) were obtained. Rarefaction curves indicated that the sequencing volume and sample depth were saturated (Supplementary Figure S1). In addition, good coverage reflects the integrity of sequencing (Wang et al., 2023). In this study, the coverage of each sample was above 99%, indicating that most of the species in the sample were detected (Supplementary Table S3).

### 3.2 Comparison of the microbial communities of the plant and the insect

**Figure 1:**
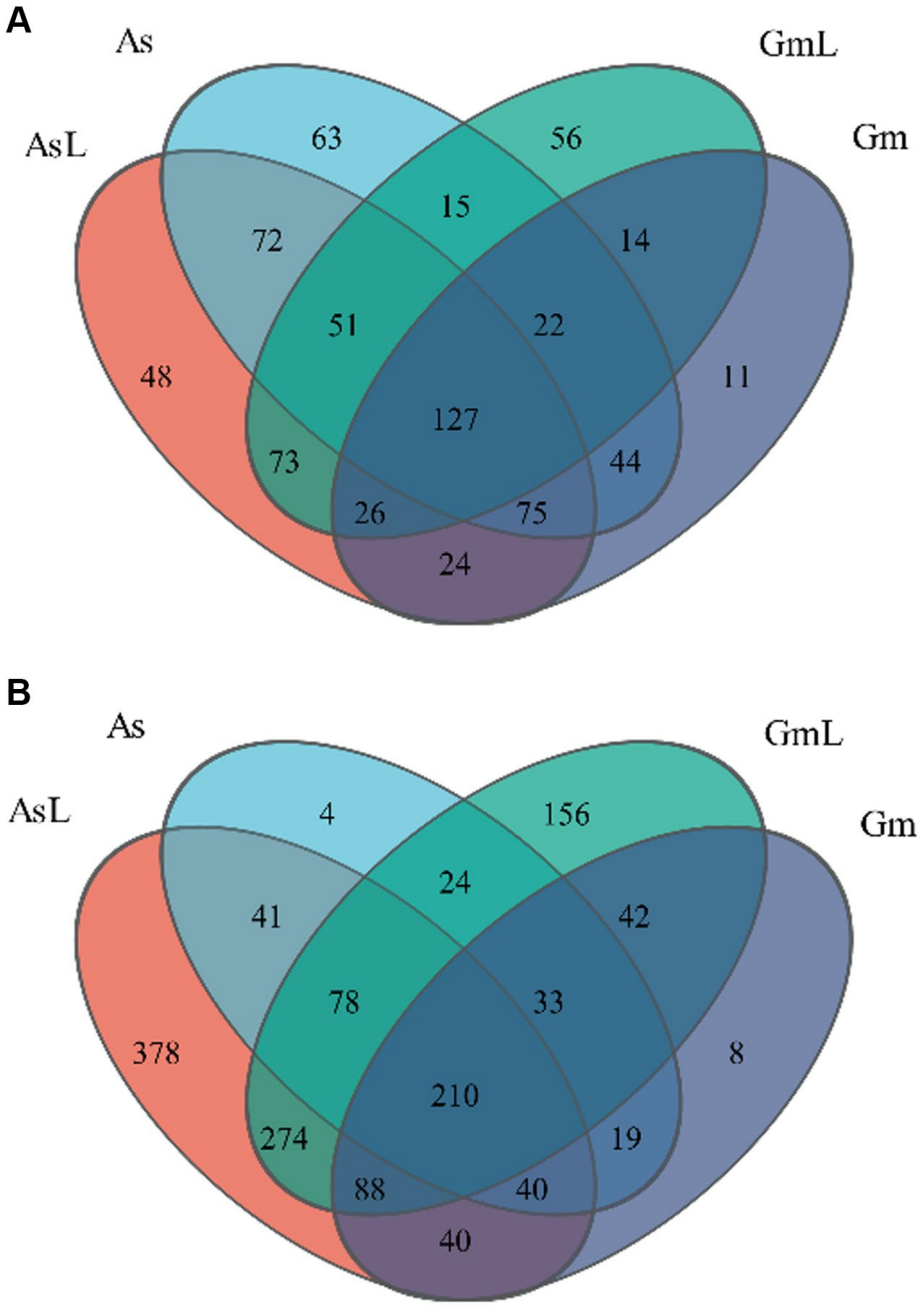
Venn diagram of the microbiota of *R. cyatheae* guts and host plant leaves. **(A)** Bacteria. **(B)** Fungi.

After high-quality filtering, the optimized sequence clustering analysis based on 97% sequence similarity assigned the high-quality bacterial sequences into 721 OTUs (544 intestinal bacterial OTUs and 603 endophytic bacterial OTUs in leaves, shared 426), belonging to 16 phyla, 24 classes, 75 orders, 132 families, 265 genera, and 417 species. Among them, 201 OTUs were observed in As, 75 were observed in Gm, and 268 were shared between groups. The analysis of OTUs in the intestine and host leaves showed that 144 OTUs were observed in As, 171 were observed in AsL, and 325 were shared between groups. Furthermore, 154 OTUs were observed in Gm, 195 were observed in GmL, and 189 were shared between groups (Figure 1A). The high-quality sequences of fungi were assigned into 1,435 OTUs (627 intestinal fungal OTUs and 1,404 endophytic fungal OTUs in leaves, shared 596), belonging to 3 phyla, 21 classes, 75 orders, 215 families, 435 genera, and 637 species. Among them, 147 OTUs were observed in As, 178 were observed in Gm, and 302 were shared between groups. The analysis of OTUs in the intestine and host leaves showed that 144 OTUs were observed in As, 171 were observed in AsL, and 325 were shared between groups. Furthermore, 154 OTUs were observed in Gm, 195 were observed in GmL, and 189 were shared between groups (Figure 1B).

**Figure 2:**
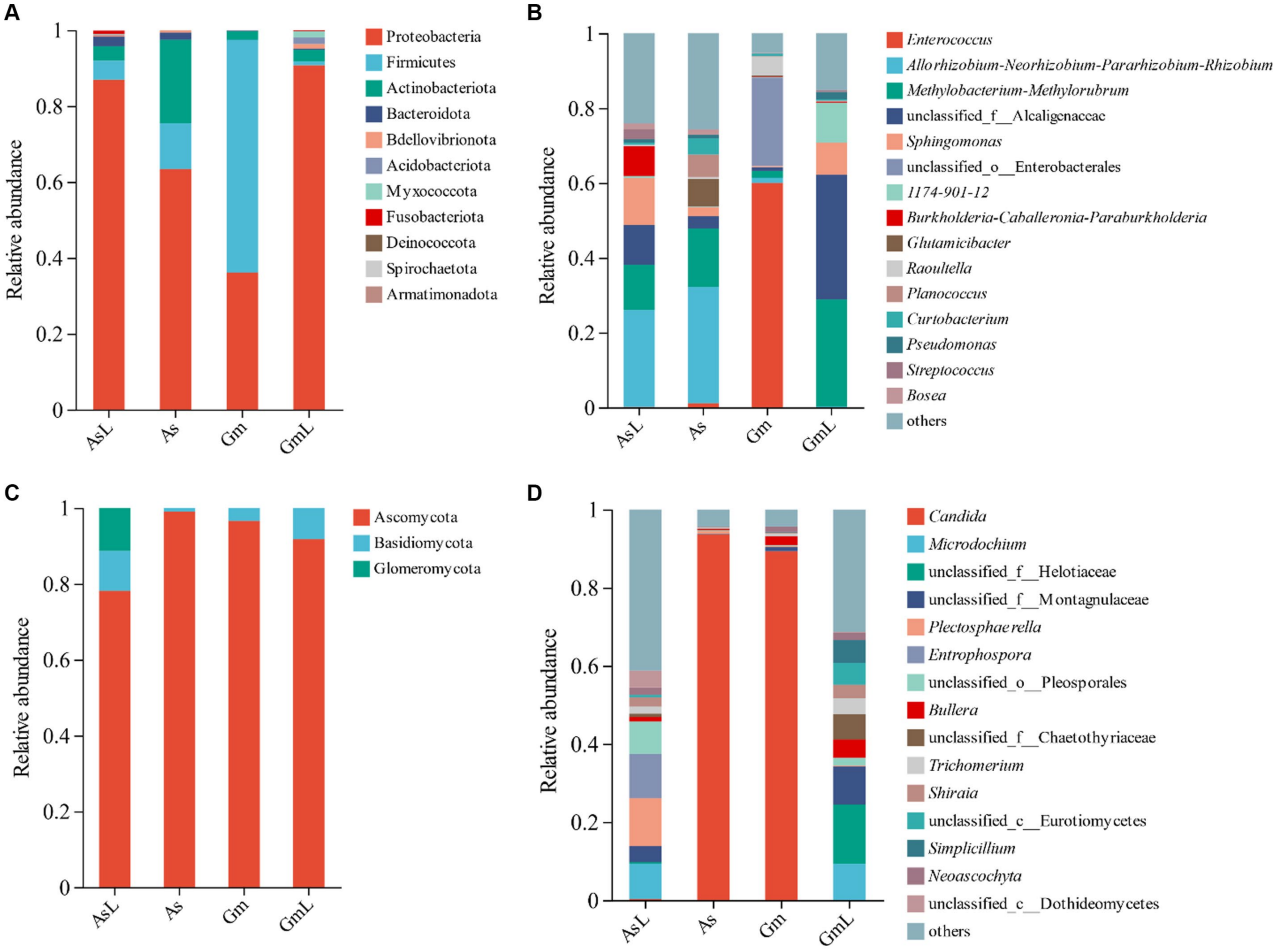
Microbial composition of *R. cyatheae* guts and host leaves. **(A)** Bacterial phylum level. **(B)** Bacterial genus level. **(C)** Fungal phylum level. **(D)** Fungal genus level.

Proteobacteria, Firmicutes, and Actinobacteria were dominant phyla in the As and Gm groups, accounting for 97.44 and 99.74% of the total sequences, respectively (Figure 2A). Proteobacteria was the dominant phylum in the AsL and GmL groups, accounting for 86.60 and 90.41% of the total sequences, respectively. At the genus level, the top five abundant genera in the gut of the two groups of *R*. *cyatheae* were different. The dominant genera in the gut of *R*. *cyatheae* feeding on *A. spinulosa* were *Allorhizobium-Neorhizobium-Pararhizobium-Rhizobium* (31.07%) and *Methylobacterium-Methylorubrum* (15.55%), which were also found in the host plants and accounted for a high proportion (25.83 and 11.96%, respectively). *Enterococcus* (59.89%) and unclassified_o_*Enterobacterales* (23.62%) were the dominant genera in the gut of *R*. *cyatheae* feeding on *G. metteniana* but were absent in GmL (Figure 2B). Collectively, the results showed that Proteobacteria, Firmicutes, and Actinobacteria were the most abundant phyla in the two hosts, whereas the dominant genera changed according to the host. At the phylum and genus levels, the similarity between the intestinal bacteria of *R. cyatheae* feeding on *A. spinulosa* and its host plant bacteria was higher than that observed in the Gm group. At the phylum level, the dominant intestinal fungus of the two feeding groups of *R. cyatheae* was Ascomycota (As: 98.98%; Gm: 96.58%). The dominant fungus in the AsL and GmL groups was also Ascomycota, accounting for 78.16 and 91.74% of the total sequences, respectively (Figure 2C). At the genus level, the dominant genus of intestinal fungi in the two feeding groups of *R. cyatheae* was *Candida* (As: 93.49%; Gm: 89.28%). However, *Candida* was not detected in the AsL and GmL groups (Figure 2D). In terms of fungal genera, the dominant genus in *R. cyatheae* was not affected by the microbiome of these two host leaves; nonetheless, the fungus exhibited its formation mechanism, which may play a special role in the intestine.

### 3.3 Diversity analysis of plant and insect microbiomes

**Figure 3:**
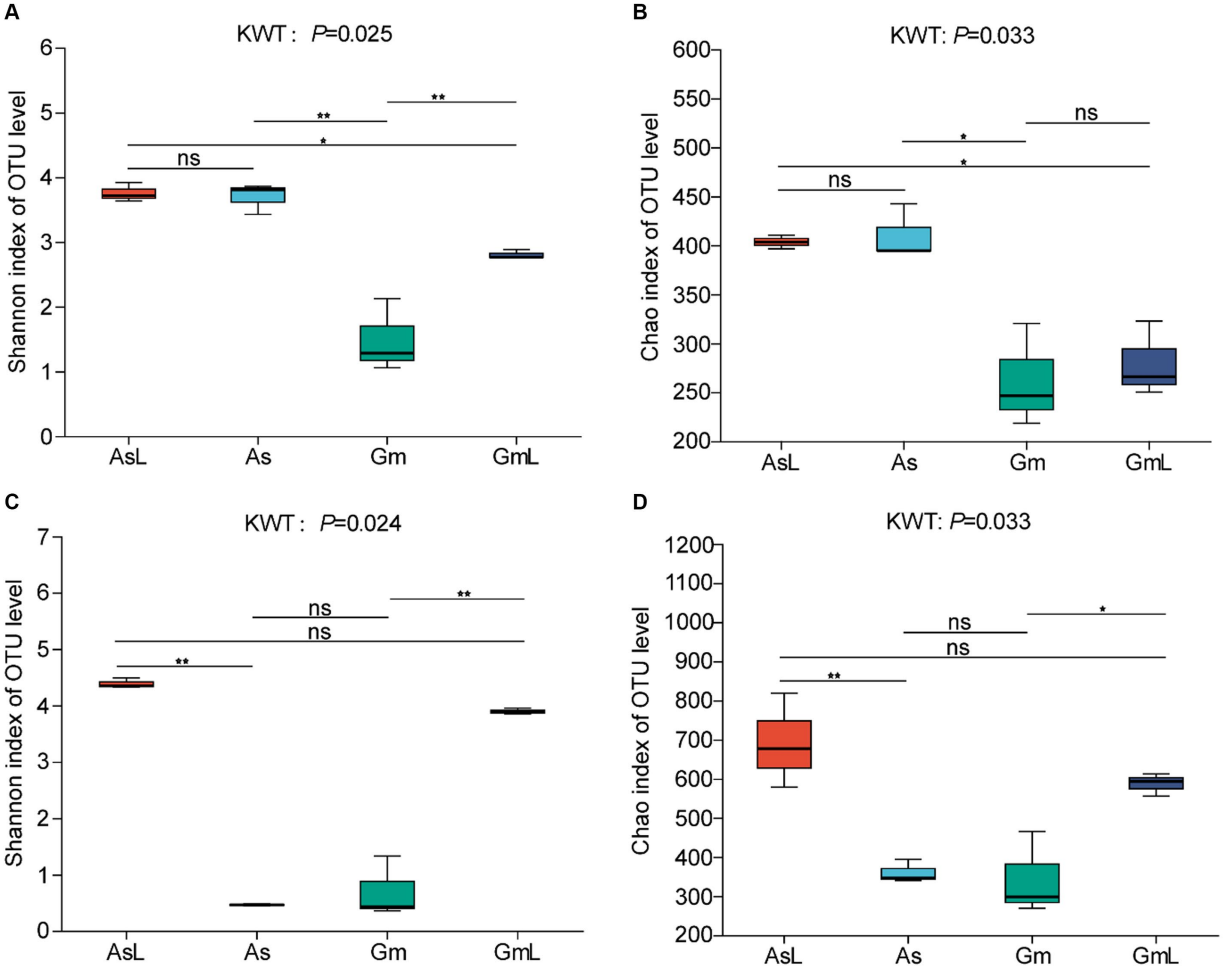
Alpha diversity of *R. cyatheae* gut and host leaf communities. Shannon diversity **(A)** bacteria; **(C)** fungi, Chao index **(B)** bacteria; **(D)** fungi. In the figure, * represents the significant difference at *p* < 0.05; ** represents the significant difference at *p* < 0.01; ns means no difference.

**Figure 4:**
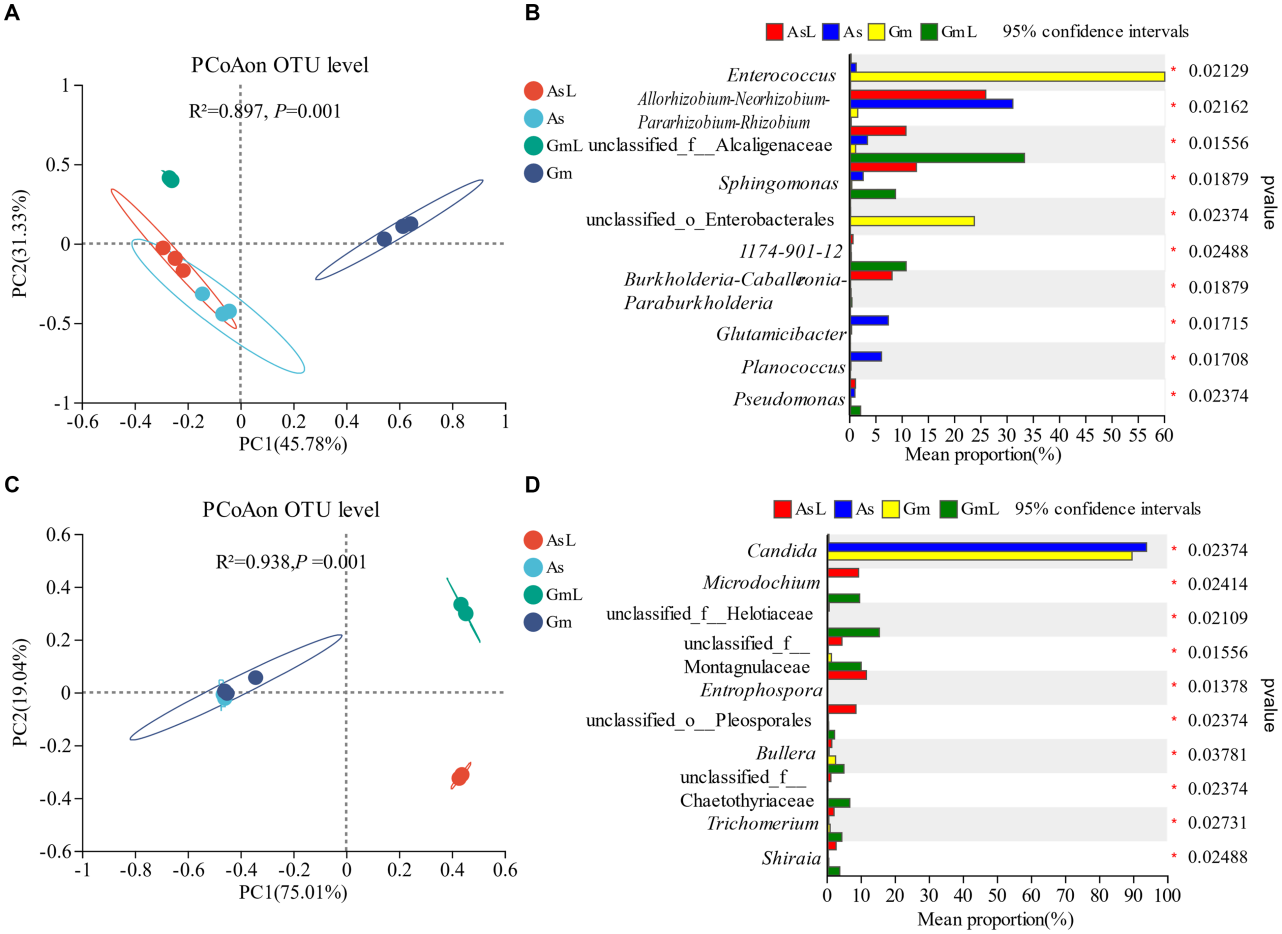
Analysis of bacteria and fungi in the intestine of *R. cyatheae* feeding on different hosts and host plants; principal coordinate analysis and genus levels. Bacteria: **(A,B)**; fungi: **(C,D)**.

Alpha diversity was used to assess the richness and diversity between groups. The intestinal bacteria of *R. cyatheae* feeding on different plants showed significant differences in Chao (Figure 3A) and Shannon indices (Figure 3B). Figure 3 shows that bacterial diversity in the intestine of *R. cyatheae* feeding on *G. metteniana* decreased significantly. For fungi, no significant difference was observed in bacterial diversity and richness in the intestine of *R. cyatheae* fed on different plants (Figures 3C,D). It is worth noting that when the Wilcoxon signed-rank test was used for pairwise comparison, we found that at the bacterial level, the Chao and Shannon indices of the gut microbiome of *R. cyatheae* were close to those of the leaves of the plants they feed on. However, significant differences were observed for fungal communities. The overall difference is explained in the principal coordinate analysis (PCoA) diagram (bacteria: Adonis, *R*^2^ = 0.897, *p* = 0.001; fungi: Adonis, *R*^2^ = 0.938, *p* = 0.001). The intestinal bacteria of *R. cyatheae* feeding on different host plants were clustered into two different quadrants. The similarity among fungi was higher than that among bacteria (Figure 4). The similarity among bacteria between the intestinal communities of *R. cyatheae* and the leaf communities of host plants was higher than that among fungi, showing a closer distance in the PCoA diagram (Figures 4A,C). To further elucidate the effects of the two host plants on the microbiota of *R. cyatheae* larvae, differential abundances of dominant genera were compared. Notably, *Enterococcus*, *Allorhizobium–Neorhizobium–Pararhizobium–Rhizobium*, unclassified_o__Enterobacterales, and *Glutamicibacter* showed differential abundances between *A. spinulosa*- and *G. metteniana*-feeding *R. cyatheae*. *Allorhizobium–Neorhizobium–Pararhizobium–Rhizobium* showed no differential abundance between *R. cyatheae* and host plants. These results indicate that host plants affected the intestinal microbiota of *R. cyatheae* larvae (Figures 4B,D).

### 3.4 Construction of the ecological network of intestinal communities

**Figure 5:**
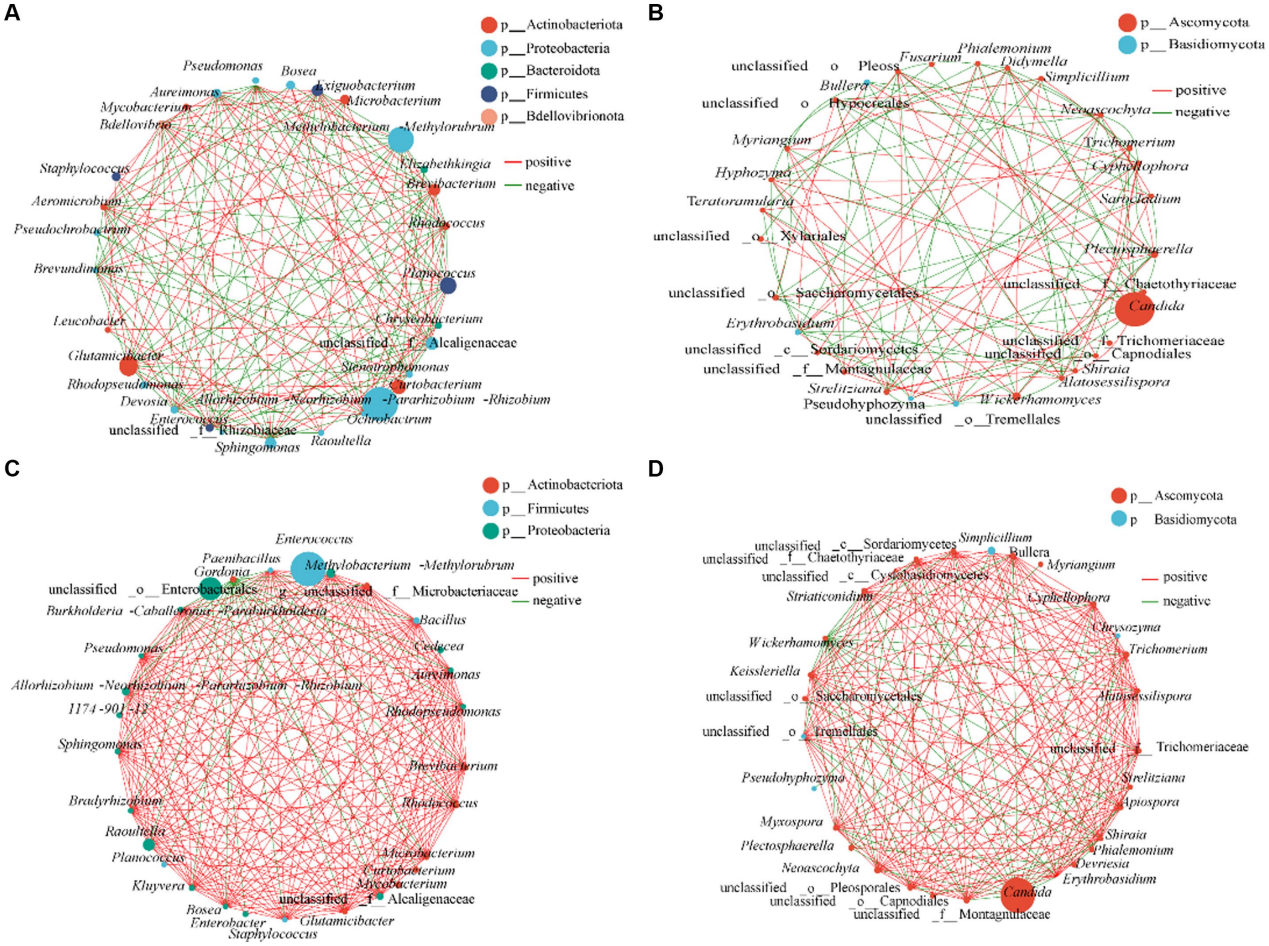
Interaction network diagram of the gut microbial genus classification level of *R. cyatheae* feeding on different host plants. **(A,B)** Intestinal bacteria and fungi of *R. cyatheae* feeding on *A. spinulosa*. **(C,D)** Intestinal bacteria and fungi of *R. cyatheae* feeding on *G. metteniana*. Nodes represent the only genus, the size of each node represents the abundance of species, the red line represents a positive correlation, and the green line represents a negative correlation. The thickness of the line indicates the size of the Spearman correlation coefficient. The figure shows that the correlation coefficient exceeds 0.6 and *p* < 0.05.

Association networks were constructed to determine the patterns of gut bacterial communities of *R. cyatheae* fed on *A. spinulosa* and *G. metteniana* (Figure 5). The network diagram of intestinal bacteria of the insect fed on *A. spinulosa* included 27 nodes and 172 edges (88 positive and 84 negative correlations) and that of intestinal bacteria of the insect fed on *G. metteniana* included 29 nodes and 223 edges (197 positive and 26 negative correlations). There were 53 positive correlations and 65 negative correlations among the fungi of *R. cyatheae* fed on *A. spinulosa*, and 159 positive correlations and 49 negative correlations among the fungi of *R. cyatheae* fed on *G. metteniana*, indicating that the complexity and pattern of the intestinal communities network structure in *R. cyatheae* fed on *G. metteniana* were higher than those in *R. cyatheae* fed on *A. spinulosa*. We found that *Allorhizobium-Neorhizobium-Pararhizobium-Rhizobium*, which showed the highest abundance in the As group, was not closely related to other bacteria in the network diagram (5 degrees), and the second dominant genus *Methylobacterium-Methylorubrum* (16 degrees) was the most closely related one. The highest abundance of *Enterococcus* was 2 degrees, and there were 22 genera with more than 16 degrees of abundance in the Gm group. Although no difference was observed in the abundance of the dominant genus *Candida* between the two groups, the degree of association with other genera in the network diagram was not the same (As: 11 degrees; Gm: 18 degrees). The above results indicated that the dominant intestinal bacteria of *R. cyatheae* fed on *A. spinulosa* were less related to other genera in life activities than those in *R. cyatheae* fed on *G. metteniana*. Overall, the results indicated that there were more cooperation and exchange events among most bacterial genera during the adaptation of *R. cyatheae* larvae to different hosts.

### 3.5 Functional prediction of gut microbiota

**Figure 6:**
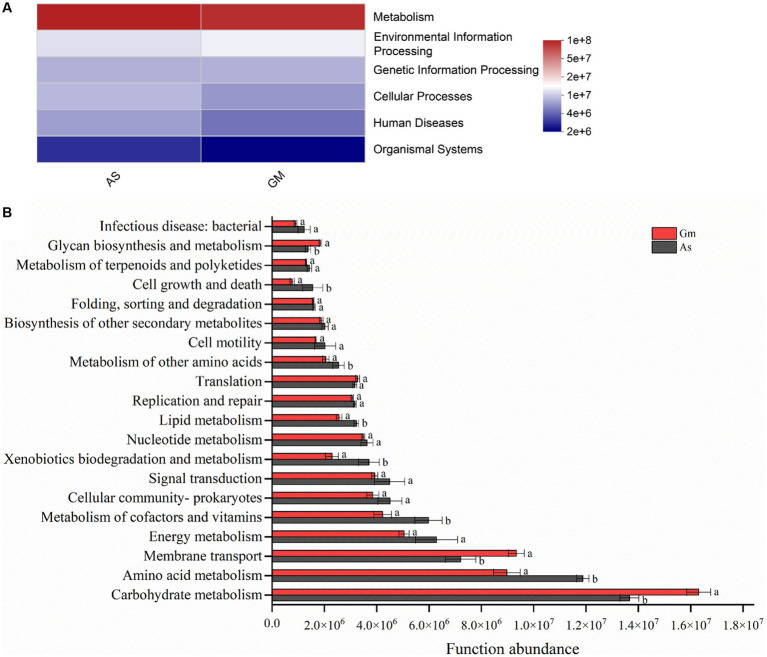
Comparison of PICRUSt2 function prediction in the gut bacterial communities of *R. cyatheae* fed on different host plants. **(A)** Heatmap of pathway level one; **(B)** analysis of metabolic pathway level two. Different lowercase letters above the bars indicate significant differences among different treatments (*p* < 0.05).

The PICRUSt2 results showed that the functional prediction categories of the gut bacteria of *R. cyatheae* feeding on different hosts were focused on metabolism, environmental information processing, genetic information processing, cell transformation, human diseases, and organismal systems. Additionally, the relative abundance of the metabolic pathway was the highest (Figure 6A). In addition, significant differences were observed in the relative abundance of the secondary classification level of metabolic pathways in each treatment (Figure 6B). Specifically, the relative abundance of carbohydrate metabolism (*p* < 0.05), amino acid metabolism (*p* < 0.05), and membrane transport (*p* < 0.05) in the gut bacteria of *R. cyatheae* feeding on *G. metteniana* was significantly higher than that of those feeding on *A. spinulosa*. The relative abundance of cofactor and vitamin metabolism (*p* < 0.05), xenobiotics symbiosis and metabolism (*p* < 0.05), and cell growth and death (*p* < 0.05) was dominant in the gut bacteria of *R. cyatheae* feeding on *A. spinulosa*.

**Figure 7:**
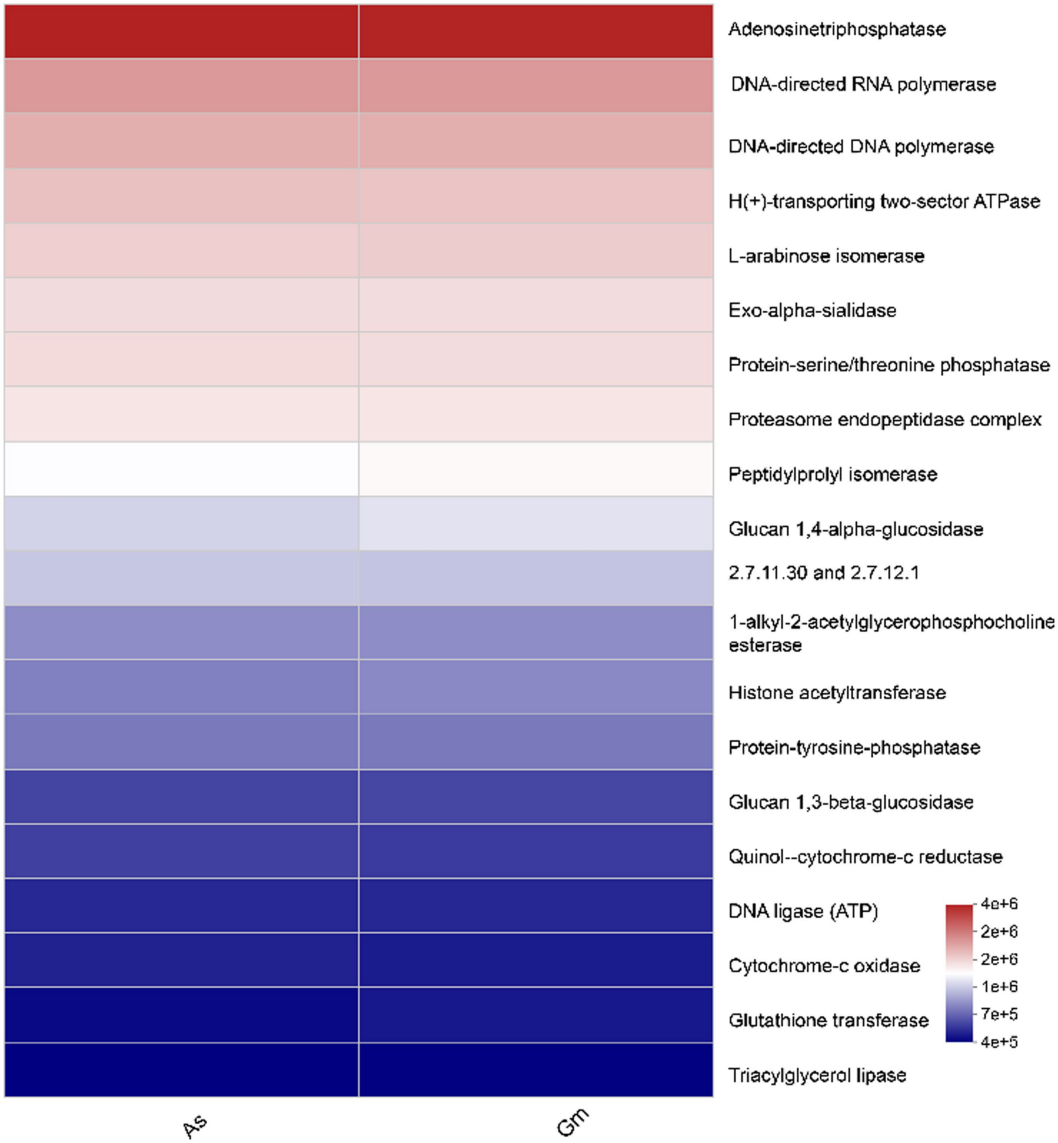
Heatmap of Kyoto Encyclopedia of Gene and Genomes function prediction of the intestinal fungi of *R. cyatheae* feeding on different hosts.

The levels of adenosine triphosphatase, DNA-directed RNA polymerase, and DNA-directed DNA polymerase were high in both groups (Figure 7). No significant difference was observed in the function of the intestinal fungi of *R. cyatheae* feeding on the two host plants.

## 4 Discussion

The gut microbiota of insects play important and diverse roles in host digestion (Engel and Moran, 2013; Jing et al., 2020), growth and development (Yang et al., 2022), detoxification (Siddiqui et al., 2022), oviposition induction (Qiao et al., 2019), and essential vitamin and amino acid production (Bisch et al., 2018). These effects enable gut microbes to enhance the adaptability of insects to the external environment, which is important for the survival and expansion of insect populations. Numerous studies demonstrated that the structure and diversity of the gut microbiota of insects could be affected by host diets (Santos-Garcia et al., 2020; Li et al., 2021). In the present study, we compared the gut microbial communities of *R. cyatheae* fed on two host plants. The Chao and Shannon indices of gut bacterial communities of *R. cyatheae* larvae fed on *A. spinulosa* were significantly higher than those fed on *G. metteniana*, which was different from the gut microbiota of overwintering *R. cyatheae* larvae measured before, indicating that environmental changes could significantly affect the bacterial structure of insects (Martemyanov et al., 2016; Zhang et al., 2023). Proteobacteria and Firmicutes dominated microbial communities of *R. cyatheae* larvae. This result was similar to the intestinal bacterial communities of many insects, such as honey bees, *Anoplophora glabripennis*, and *Spodoptera frugiperda* (Warnecke et al., 2007; De Oliveira Scoaris et al., 2021; Wang et al., 2022; Han et al., 2023; Wang et al., 2023). The dominant bacteria of the two groups of *R. cyatheae* were significantly different at the genus level. *Allorhizobium-Neorhizobium-Pararhizobium-Rhizobium* is a common bacterial genus in roots and leaves, which accounts for a relatively high proportion in the intestines of *R. cyatheae* fed *on A. spinulosa*, and may enter the intestines via the leaves to become resident or transit bacteria. *Enterococcus*, which dominated the gut of *R. cyatheae* feeding on *G. metteniana*, has been found in *Helicoverpa armigera*, honey bees, and *Spodoptera littoralis*, and it is a dominant genus in many insects. It plays an active role in insect host adaptation, primarily by synthesizing amino acids and vitamins, degrading secondary compounds and cell walls, regulating intestinal pH, and enhancing intestinal immunity (Ruiz-Rodriguez et al., 2012; Dantur et al., 2015; Vilanova et al., 2016; Shao et al., 2017; De Oliveira Scoaris et al., 2021; Li et al., 2022). We speculate that *Enterococcus* may play a role in the adaptability of *R. cyatheae* to *G. metteniana*, which may be caused by different plant secondary compounds in the leaves of the two hosts. It is necessary to adjust the intestinal microorganisms to absorb energy more effectively when the insect is feeding on *G. metteniana*.

We found no difference in the dominant phylum of intestinal fungi in the insect fed on the two host plants. Ascomycota dominated the two groups, which was consistent with the results of the intestinal fungi of *Bombyx mori*, aphid, and *A. glabripennis* (Chen et al., 2018; Mason et al., 2019b; Wolfgang et al., 2023). *Candida* was the dominant genus in the two groups, which is widespread in the environment and causes most fungal infections worldwide (Silva et al., 2012). *Candida* has been isolated from the intestines of *Agrilus mali*, *Dendroctonus armandi*, and other insects (Hu et al., 2015; Zhang et al., 2018). *Candida* produces xylanase and lipase, which are involved in adipose tissue decomposition and lipoprotein degradation in host life. It converts oil into free fatty acids and partially acyl glycerol (Suh and Blackwell, 2004; Hu et al., 2015; Zhang et al., 2018). As the dominant intestinal fungus of *R. cyatheae*, the function of *Candida* is still unclear and needs further investigation.

Plant and herbivory insect microbiomes are in a dynamic two-way interaction (Wolfgang et al., 2023). Insects consume plant materials and endogenous microorganisms as food, shaping the microbial community of the insect gut, which can be linked to the ability of insects to defeat plant defenses (Montagna et al., 2015; Pirttilä et al., 2023). Gut microbes can transfer genes to insects via horizontal gene transfer and provide enzymes to digest plant materials to help the insects quickly adapt to host plants (Hansen and Moran, 2014). In the present study, we found that feeding on different host plants affected the gut microbiota of *R. cyatheae*. We found no difference in the abundance of *Allorhizobium-Neorhizobium-Pararhizobium-Rhizobium* between AsL and *R. cyatheae* (Welch T-test: *p* > 0.05) (Wolfgang et al., 2023) found that *Allorhizobium-Neorhizobium-Pararhizobium-Rhizobium* existed in soil, leaves, and aphids. However, we found that this shared microorganism was less abundant in *R. cyatheae* fed on *G. metteniana*, and bacteria with high abundance in the gut of *R. cyatheae*, such as *Enterococcus*, were rare in the leaves of *G. metteniana* (Welch T-test: *p* < 0.05). It indicates the similarities between the microbiomes of insect guts and host leaves, and the extent of the overlap between the plant and insect microbiomes is likely highly dependent on species specificity (Pirttilä et al., 2023). Therefore, we concluded that the similarity between the intestinal bacteria of the GmL and *R. cyatheae* was lower than the similarity between the intestinal bacteria of the AsL and *R. cyatheae*. The intestinal fungi were less affected by leaf endophytes, and no significant difference was observed between the two groups. This may be related to the different metabolites of leaves. The habit of feeding on different nutrients or potentially toxic substances may help the insect effectively control the intestinal microbial species and rapidly degrade and digest these substances, thus helping the insect better adapt to different environments (Meriweather et al., 2013; Šigutová et al., 2023). Besides defeating plant defenses and supporting exploitation by the insect herbivore, the transmitted microbes can alter the survival, fecundity, and immunity of the insect host and can therefore alter the fitness of the herbivore (Saikkonen et al., 1996; Mauck et al., 2016) found that the relative growth rate of larvae was lower and the larval stage was longer when feeding on plants with higher mean endophytic bacteria abundances. We also found significant differences in the growth and development of *R. cyatheae* fed on the two host plants (Supplementary Table S5). The association network revealed that different hosts affected the microbial networks. The higher numbers of network topology properties, such as the number of nodes, positive correlations, negative correlations, and degree observed in the group feeding on *G. metteniana* indicated a complex network for this group. The results showed that feeding on *G. metteniana* was superior at enhancing the complexity of the intestinal microbial network and the resistance of the system to the external environment, which was consistent with the higher survival rate of *R. cyatheae* fed on *G. metteniana* by Xiao et al. (2023). The PICRUSt2 results showed that the functional prediction of the gut bacteria of *R. cyatheae* feeding on *G. metteniana* was more closely related to carbohydrate and amino acid metabolism, which could better provide nutrition for host insects and enhance the adaptability of the hosts.

## 5 Conclusion

This study highlights the importance of the effect of diet on the structure of intestinal microbial communities of *R. cyatheae*. The intestinal microbial communities of *R. cyatheae* fed on different host plants were diverse, and the dominant bacteria were also different accordingly. After feeding on different diet, the intestinal fungi of *R. cyatheae* showed a more stable community structure than that of the intestinal bacteria. There were more events of cooperation and communication among gut microbes of *R. cyatheae* feeding on *G. metteniana*. Altogether, this study provides a theoretical basis for further understanding the adaptability of *R. cyatheae* to host plants.

## Data availability statement

The raw data supporting the conclusions of this article will be made available by the authors, without undue reservation.

## Ethics statement

Ethical approval was not required for the study involving animals in accordance with the local legislation and institutional requirements because the research object is small herbivorous insects, and the number is small and does not involve ethics.

## Author contributions

BZ: Investigation, Writing – original draft, Writing – review & editing. WY: Funding acquisition, Project administration, Resources, Writing – review & editing. QH: Funding acquisition, Supervision, Writing – review & editing. HC: Project administration, Writing – original draft, Writing – review & editing. BC: Data curation, Validation, Writing – review & editing. XB: Conceptualization, Investigation, Writing – review & editing.

## Footnotes

^1^https://github.com/OpenGene/fastp, version 0.19.6.

^2^http://www.cbcb.umd.edu/software/flash, version 1.2.11.

^3^http://huttenhower.sph.harvard.edu/LEfSe

^4^http://huttenhower.sph.harvard.edu/galaxy
