# Supplementary material for: Analysis of differential effects of host plants on the gut microbes of Rhoptroceros cyatheae
Source: Front Microbiol. 2024 Jun 19;15:1392586. doi: 10.3389/fmicb.2024.1392586 (PMC11221597; doi:10.3389/fmicb.2024.1392586)
Supplement: Supplementary file 1 [file Data_Sheet_1.doc]

Supplementary Material

Table S1: 16S rDNA sequencing analysis of intestinal bacteria of *R.cyatheae* and host leaves

| Sample | Sequences number(bp) | Base number(bp) | Mean_length(bp) | Min_length(bp) | Max_length(bp) |
| --- | --- | --- | --- | --- | --- |
| AsL_1 | 66225 | 24891159 | 375.857441 | 205 | 432 |
| AsL_2 | 76109 | 28609344 | 375.899618 | 214 | 405 |
| AsL_3 | 78427 | 29471400 | 375.7813 | 206 | 408 |
| As_1 | 73200 | 27594273 | 376.970943 | 212 | 407 |
| As_2 | 81607 | 30803916 | 377.46659 | 214 | 479 |
| As_3 | 79780 | 30129404 | 377.656104 | 214 | 525 |
| GmL_1 | 93178 | 34955793 | 375.150712 | 212 | 516 |
| GmL_2 | 73940 | 27763340 | 375.484717 | 212 | 432 |
| GmL_3 | 72653 | 27298454 | 375.737464 | 210 | 489 |
| Gm_1 | 43875 | 16551363 | 377.239043 | 204 | 400 |
| Gm_2 | 43612 | 16448424 | 377.153627 | 361 | 402 |
| Gm_3 | 49192 | 18559319 | 377.283278 | 318 | 498 |

Table S2: ITS sequencing analysis of intestinal fungi of *R.cyatheae* and host leaves

| Sample | Sequences number(bp) | Base number(bp) | Mean_length(bp) | Min_length(bp) | Max_length(bp) |
| --- | --- | --- | --- | --- | --- |
| AsL_1 | 96930 | 24360925 | 251.324925 | 200 | 528 |
| AsL_2 | 99693 | 25054279 | 251.314325 | 200 | 534 |
| AsL_3 | 97393 | 24323100 | 249.741768 | 200 | 447 |
| As_1 | 100404 | 25645746 | 255.425541 | 200 | 457 |
| As_2 | 74612 | 19007115 | 254.746086 | 200 | 515 |
| As_3 | 75298 | 19231639 | 255.407036 | 200 | 392 |
| GmL_1 | 102942 | 25098873 | 243.815673 | 200 | 494 |
| GmL_2 | 99009 | 24366310 | 246.101971 | 200 | 518 |
| GmL_3 | 99069 | 24279217 | 245.073807 | 200 | 514 |
| Gm_1 | 71575 | 18250107 | 254.978791 | 200 | 382 |
| Gm_2 | 79346 | 20006095 | 252.137411 | 200 | 489 |
| Gm_3 | 78359 | 19974926 | 254.91553 | 200 | 450 |

Table S3: The α diversity index of intestinal bacteria of *R.cyatheae* and host leaves

| Sample | sobs | shannon | simpson | ace | chao | coverage |
| --- | --- | --- | --- | --- | --- | --- |
| ASL1 | 379 | 3.62953 | 0.07661 | 400.098858 | 396.33333 | 0.998995 |
| ASL2 | 378 | 3.91434 | 0.05478 | 402.898412 | 410.25000 | 0.998920 |
| ASL3 | 381 | 3.71011 | 0.05671 | 400.470074 | 403.28571 | 0.998995 |
| AS1 | 333 | 3.42183 | 0.08115 | 383.539414 | 394.17647 | 0.998367 |
| AS2 | 368 | 3.80324 | 0.05386 | 396.883241 | 394.36585 | 0.998819 |
| AS3 | 383 | 3.85822 | 0.05147 | 417.439237 | 442.23077 | 0.998593 |
| GML1 | 297 | 2.76354 | 0.19022 | 313.266301 | 322.50000 | 0.999146 |
| GML2 | 258 | 2.87919 | 0.16435 | 267.884519 | 265.77273 | 0.999523 |
| GML3 | 240 | 2.75274 | 0.13273 | 249.814264 | 250.00000 | 0.999498 |
| GM1 | 285 | 2.12153 | 0.28395 | 330.676297 | 320.17021 | 0.998543 |
| GM2 | 213 | 1.27904 | 0.48727 | 248.279037 | 246.17647 | 0.998794 |
| GM3 | 297 | 2.76354 | 0.19022 | 313.266301 | 322.50000 | 0.999146 |

Table S4: The α diversity index of intestinal fungi of *R.cyatheae* and host leaves

| Sample | sobs | shannon | simpson | ace | chao | coverage |
| --- | --- | --- | --- | --- | --- | --- |
| ASL1 | 784 | 4.323937 | 0.045734 | 799.207785 | 818.18182 | 0.999189 |
| ASL2 | 577 | 4.342436 | 0.042111 | 578.499010 | 578.00000 | 0.999882 |
| ASL3 | 673 | 4.484950 | 0.033787 | 676.053861 | 676.25000 | 0.999764 |
| AS1 | 275 | 0.441353 | 0.884084 | 400.792015 | 393.40426 | 0.998210 |
| AS2 | 253 | 0.474719 | 0.866480 | 347.987904 | 346.06818 | 0.998463 |
| AS3 | 271 | 0.460735 | 0.872742 | 362.908607 | 339.25000 | 0.998463 |
| GML1 | 601 | 3.851426 | 0.064321 | 610.827854 | 612.07143 | 0.999476 |
| GML2 | 570 | 3.874524 | 0.050167 | 586.114559 | 592.57500 | 0.999274 |
| GML3 | 546 | 3.950970 | 0.054453 | 554.948363 | 554.78378 | 0.999561 |
| GM1 | 218 | 0.355233 | 0.908489 | 282.036974 | 268.62222 | 0.998852 |
| GM2 | 404 | 1.325510 | 0.616022 | 468.676494 | 464.68182 | 0.998480 |
| GM3 | 237 | 0.426425 | 0.888954 | 308.898816 | 297.63830 | 0.998716 |

Table S5: Growth and development indexes of *R.cyatheae* feeding on different hosts

| Host | *A. spinulosa* | *G. metteniana* |
| --- | --- | --- |
| Egg stage (d) | 4.22 ± 0.45a | 4.03 ± 0.32b |
| Larvae stage (d) | 10.48 ± 0.72a | 10.35 ± 0.66a |
| Mature larvae (d) | 5.87 ± 0.70a | 5.63 ± 0.66a |
| Pupa stage (d) | 4.68 ± 0.68a | 4.05 ± 0.50b |
| Male adult stage (d) | 6.63 ± 1.30a | 6.30 ± 1.09a |
| Female adult stage (d) | 9.97 ± 1.99a | 9.57 ± 1.94a |
| Egg hatchability (%) | 85.63 ± 4.27a | 87.50 ± 3.54a |
| Larvae survival rate (%) | 45.98 ± 4.32b | 66.34 ± 4.98a |
| Eclosion rate (%) | 87.42 ± 4.84a | 89.24 ± 2.23a |
| Pupal weight (mg) | 59.59 ± 18.64a | 50.41 ± 13.26b |
| Fecundity(egg/female) | 77.88 ± 4.46a | 71.92 ± 2.21a |

Note: Data in the table were mean ± SD. Different letters in the same column indicated significant difference (*P*<0.05).


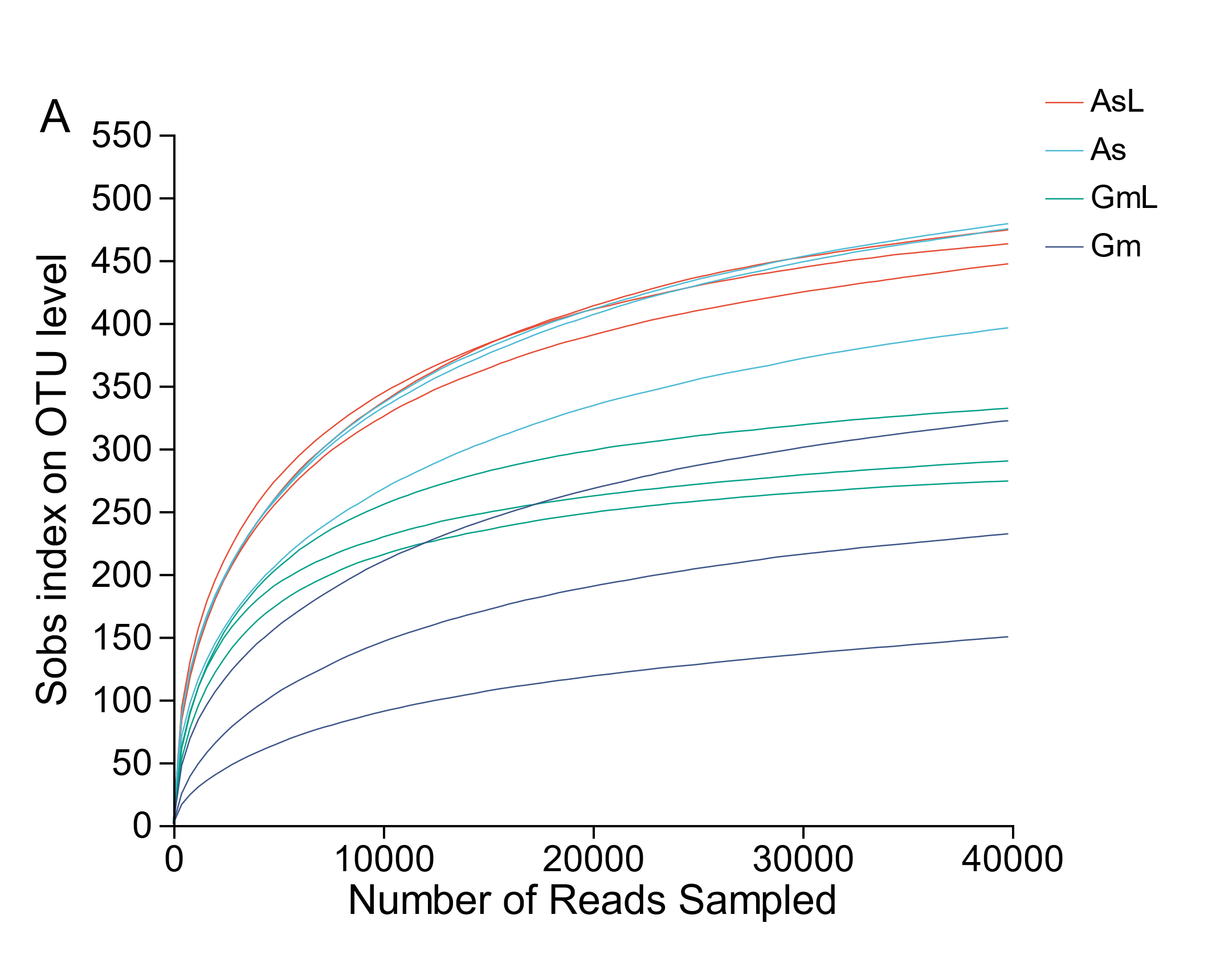

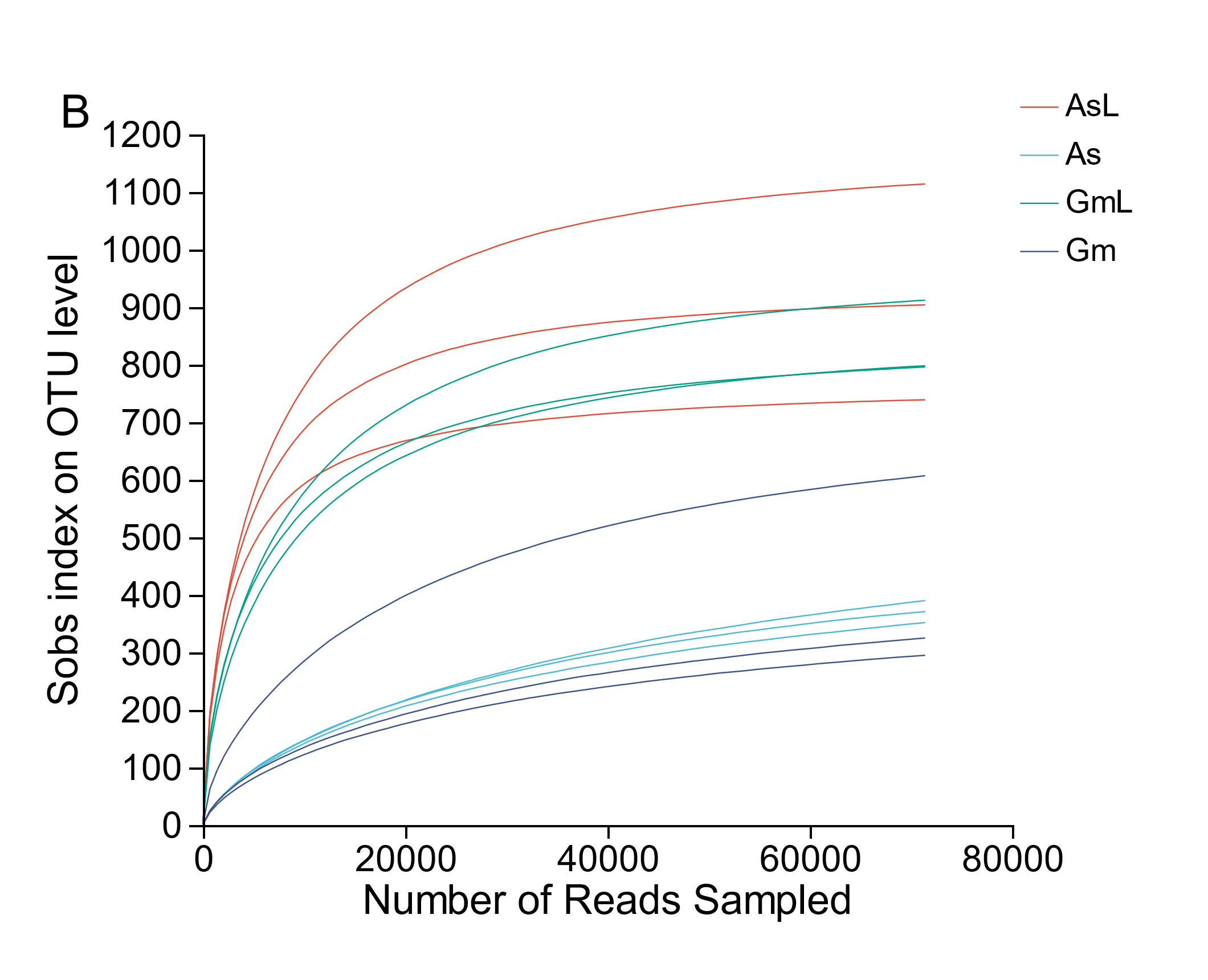


Fig S1: The α diversity index Rarefaction curves of bacteria and fungi in the intestinal of *R. cyatheae* feeding on different hosts and host leaves (A: Bacteria; B: Fungi)


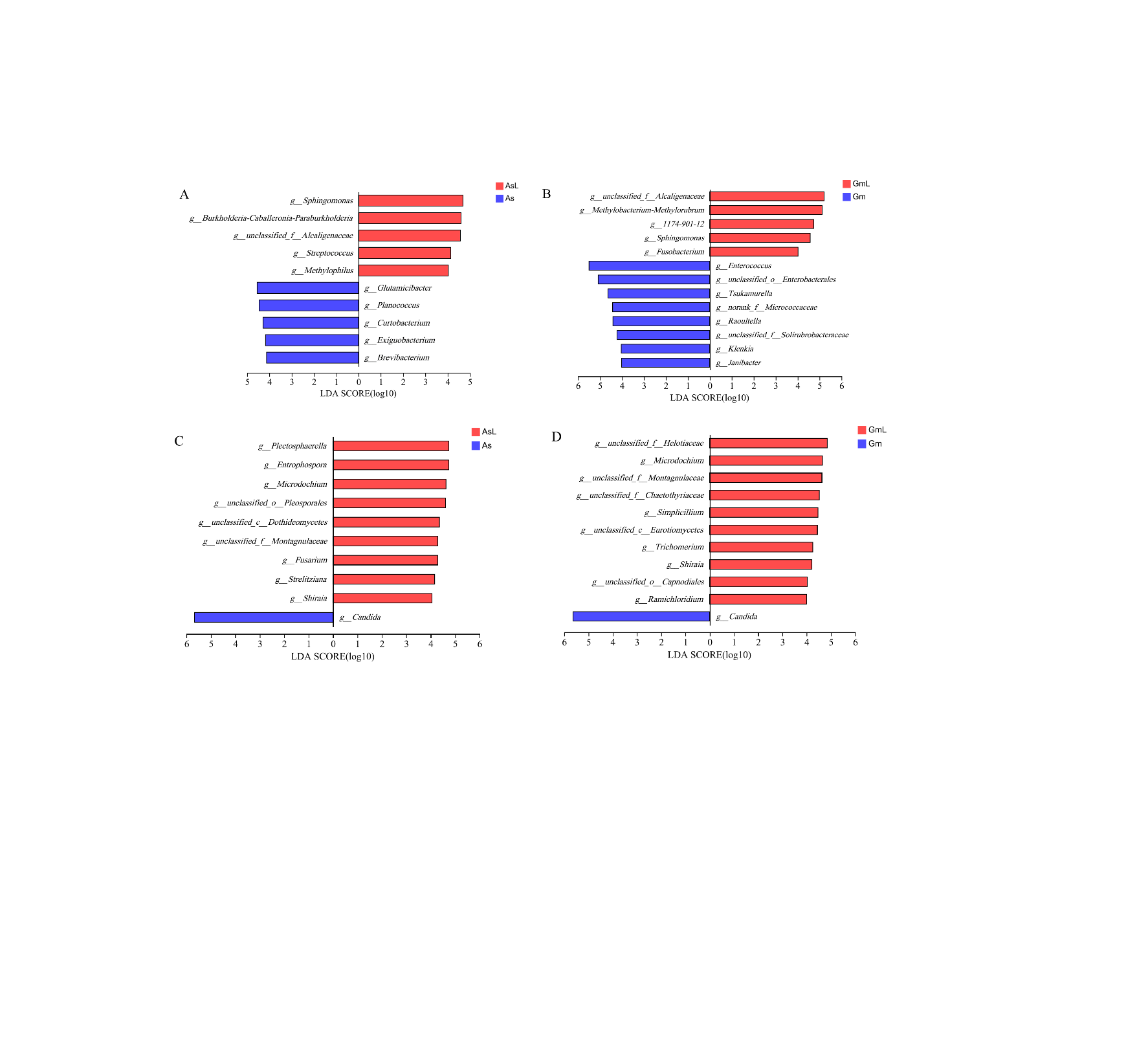


Fig S2: LDA value distribution histogram of gut microbes *R.cyatheae* and leaf microbes. Comparison of microbial differences between the leaves of *A.spinulosa* and the gut of *R.cyatheae* (A: bacteria; B: Fungi); comparison of microbial differences between the leaves of *A. spinulosa* and the gut microbes of *R.cyatheae* (C: bacteria; D: Fungi)
